# Proceedings of the Cook County Medical Society

**Published:** 1857-05

**Authors:** 


### MEDICAL INTELLIGENCE.

*Proceedings of the Cook Co. Med. Society, at the Monthly Meeting on the first Tuesday in March,* 1857. *Diseases of the Heart and Pericardium.*

The Society met at the residence of Dr. W. W. Allport. After reading the minutes of the previous meeting and the election of new members, Dr. N. S. Davis reported three cases of Cardiac disease, and presented to the Society a very interesting pathological specimen.

*Case I.*—M. B———, a German, aged about 25 years, a laborer, was admitted to the medical wards of the Mercy Hospital with severe symptoms of Acute Rheumatism. He had been attacked with the disease two weeks previous to his admission.

At the time he was brought under my care, his skin was hot; tongue covered with a white fur and dry; pulse 110 per minute and firm under pressure; nearly all the articulations both in the upper and lower extremities, were moderately swollen and slightly red; his position was dorsal and fixed. The patient complained of much thirst, and constant pain in his dorsal spine of a very severe character, with paroxysms of excruciating pain in both lower extremities. The slightest motion, or even a moderate jar of the bedstead would cause him to cry out with pain. There seemed to be a morbid increase of sensibility throughout the whole cutaneous surface. Percussion did not indicate cardiac dulness over a larger space than natural; but the impulse of the heart was quick, the first sound loud and ringing, though without distinct valvular roughness or murmurs; and it was accompanied by a plain pericardial friction sound a little below and between the nipple and the sternum. The phenomena of this case plainly indicate not merely acute articular rheumatism, but in addition thereto an unusual irritation of the central portion of the spinal system of nerves, and active pericarditis. The first is indicated by the severe pain in the spine, the intense suffering occasioned by motion or even jarring of the bed, and the general morbid sensibility of the whole system. The special seat of this irritation is probably the fibrous sheaths of the nerves at their exit from the spinal canal; and is of the same nature with the rheumatic inflammation in the extremities. The pericarditis is indicated with still greater certainty by the physical signs; and from the continuance of the *friction* sound without any material increase in the extent of the cardiac dulness, we may infer that the pericardial inflammation is still in its first stage unattended by serous effusion. If such was the pathological condition of the patient at that time it is easy to see the tendency to certain changes of importance during the further progress of the case: of these, effusions, either plastic or serous, are of chief importance. The first will be most apt to occur in the fibrous tissues surrounding the joints, and the latter in the cavity of the pericardium. Hence the indications for treatment were three-fold, viz.: 1st, To mitigate the sufferings of the patient by anodynes; 2nd, To arrest the local inflammatory actions; and 3d, To prevent, or at least to limit very much the resulting effusions.

To accomplish these purposes, the patient was directed to take the following, viz.:—

℞—Sat. Tinct. Verat. Viride, . . ʒj.

Vin. Colchici. . . . . . . ℥ss.

Tinct. Opii et Camph. . . . ℥jss.

Mix, and take one teaspoonful every four hours in sweetened water.

℞—Sub. Murias Hydrarg, . . 12grs.

Pulv. Doveri, . . . . . ℈ij.

Nit. Potassa, . . . . . ℈ij.

Mix, and divide into 6 powders. Take one between each time of taking the veratrum mixture. At the end of twenty-four hours after commencing these remedies, the febrile action was much reduced. The pulse was 80 per minute, and soft; the skin covered with perspiration; the pains, though still considerable, were much less than on the day previous. But the colchicum and veratrum mixture operated too freely on the bowels, and the former was omitted.

On the third day after admission the pericardial friction sound ceased, but the extent of dulness was much increased, the beathing was short and oppressed, and the impulse and sounds of the heart appeared more distant from the ear. It was evident that serous effusion had taken place into the pericardial sac.

The patient continued the use of alterative doses of mercurials in conjunction with anodynes and sedatives, blisters to the chest and spine, and alkaline drinks, until the gums showed a slight influence from the mercurial, when it was omitted and the other remedies continued, with the addition of a full dose of opium, quinine, and nitrate of potassa every night at bedtime. In two weeks the external or articular pains and inflammation had ceased; the indications of serous effusion had also disappeared, and a modified *friction* sound was again audible. At the end of the third week the patient had so far recovered as to leave the hospital.

*Case II.*—The second case was an Irish laborer, aged 25 years.

He had had acute or sub-acute rheumatism about three years previous, from which he apparently recovered in two or three months. During the last eighteen months he has been troubled with shortness of breath, inability to take active exercise, dull pain in the cardiac region, uneasiness, and disposition to cough on assuming the recumbent posture, and at times palpitations of the heart. At the time of his admission to the hospital, he had no fever or rheumatic pains, and his nutrition was good. But, in addition to the symptoms above-named, his pulse was strong, full, and ninety per minute; the impulse of the heart forcible, giving an unusual motion to the walls of the chest. Percussion showed a decided cardiac dulness over a larger space than normal; and auscultation revealed a loud, rough, or rasping bellows murmur over the base of the heart, without any interval between the first and second sound. It was evident that this case, which doubtless in the beginning was simple rheumatic endocarditis, now consisted in permanent thickening of the valves of the aorta with much hypertrophy of the ventricles. From the length of time which had elapsed since its commencement, there could be little doubt but the changes in the structure of the heart had become organic and incurable. Still much might be done to ameliorate the condition of the patient and prolong life by the judicious use of sedatives, to lessen the violence of the heart's action and thereby retard the abnormal growth, and by carefully avoiding all excitement, either mental or physical. We gave the patient saturated tincture of veratrum viride in doses of four drops, with spts. nit. dulc. and water every four hours; and three times a-day one of the following pills, viz.:—

| | |
|---|---|
| ℞—Acetas Plumbi, . . | 20grs. |
| Pulv. Digitalis Leaves, | 20grs. |
| Pillulæ Hydrarg. . | 20grs. |

Mix and divide into 20 pills.

Under this treatment the heart's action was much diminished, the patient could assume the recumbent posture with much less inconvenience, and at the end of three weeks felt so much more comfortable that he left the hospital; though the physical signs of valvular disease and hypertrophy still continued with but little change.

*Case III.*—A German, aged 20 years; had been sick more or less for six or seven years. He was somewhat emaciated; extremities cool; skin, especially on the cheeks and prolabia, of dark veinous or purplish color; the breathing oppressed and difficult; frequent cough, with some purulent expectoration; pulse one hundred per minute, quick, but soft; bowels regular; appetite much impaired; and incapable of any muscular exertion. Percussion showed marked dulness over nearly the whole of the left side of the chest, from the clavicle to the diaphragm, and from the sternum to the spine; but there was no apparent enlargement of that side. Auscultation gave cavernous respiration and voice in the infra-clavicular region of left side, and a loud, sub-crepitant ronchus over nearly all the rest of the left lung. The impulse of the heart was quick but weak, and accompanied by a loud, rasping, valvular murmur, with slight regurgitation. The pulmonary circulation in this patient was extremely feeble, the slightest exertion, or the depressing influence of a cathartic would cause great exhaustion with complete cyanosis.

*Diagnosis*—Thickening of the aortic valves and hypertrophy of the heart, with dilitation, and extensive tubercular disease of the left lung.

This patient was rendered much more comfortable, and his blood more perfectly arterialized by a solution of common salt, quinine, and morphine, in such proportion that he took fifteen grains of the first, one grain of the second, and a quarter of a grain of the third every four hours. After remaining in the hospital about four weeks, he died suddenly in the night. A *post mortem* examination showed tubercular deposits in both lungs, with a large cavity at the apex of the left. The heart was enlarged to twice the normal size, as members of the Society may see by examining it, which I hold in my hand. The pericardium is closely adherent throughout its whole extent to the heart by false membrane of old formation; the left ventricle is greatly dilated and its walls thick, and both the semilunar and mitral valves are very much thickened and almost of a cartilaginous hardness; the right ventricle is of normal size and valves healthy, but its walls are atrophied in a marked degree.

The foregoing report is made up from notes of clinical lectures given to the class attending the hospital during the winter term of Rush Medical College. The cases were freely and repeatedly examined by the class, and also the morbid specimens from Case III.

